# Analyzing characteristics of collateral flow to parasylvian cortical arteries by three-dimensional digital subtraction angiography–magnetic resonance angiography fusion imaging in adult moyamoya disease

**DOI:** 10.3389/fneur.2023.1251844

**Published:** 2023-09-21

**Authors:** Jin Yu, Miao Hu, Jianjian Zhang, Jincao Chen

**Affiliations:** Department of Neurosurgery, Zhongnan Hospital of Wuhan University, Wuhan, China

**Keywords:** moyamoya disease, cerebral blood flow, hemodynamics, anterior cortical circulation, bypass surgery

## Abstract

**Objective:**

The hemodynamic sources of recipient parasylvian cortical arteries (PSCAs) were significantly related to postoperative cerebral hyperperfusion (CHP) after bypass surgery in patients with moyamoya disease (MMD). The present study aimed to introduce a new method to investigate the characteristics of PSCAs hemodynamic sources and their relationships with clinical presentations in adult MMD and to provide preoperative evaluation for recipient vessel selection in MMD bypass surgery.

**Methods:**

The hemodynamic sources of the PSCAs in 171 symptomatic MMD hemispheres were analyzed by three-dimensional digital subtraction angiography (3D-DSA) combined with magnetic resonance angiography (MRA) fusion imaging. The spatial and temporal characteristics of the hemodynamic sources of the PSCAs and their associations with the patient's demographics, Suzuki stage, and initial onset type were investigated.

**Results:**

Six major types of hemodynamic sources in the PSCAs were observed. There was a significant difference between the hemodynamic sources of the PSCAs above and below the SF (*P* < 0.001). With advancing Suzuki stages, collateral flow to the PSCAs above the SF from the internal carotid arteries (ICAs) significantly decreased, while the non-ICAs increased (*P* < 0.001). Multivariate analysis revealed that hemodynamic sources of the PSCAs above the SF were significantly associated with patients' initial onset type (*P* = 0.026).

**Conclusion:**

In MMD hemispheres, the hemodynamic sources of the PSCAs above the SF are more varied than those below the SF and present a typical conversion trend from ICAs to non-ICAs with advancing Suzuki stages. Analyzing the hemodynamic sources of the PSCAs can help in understanding the conversion pattern of compensatory vascular systems, predicting episodes in MMD, and preoperatively evaluating suitable recipient vessel selection for bypass surgery to avoid postoperative CHP.

## Introduction

During the development of moyamoya disease (MMD), numerous compensatory vascular systems are established to fight against reduced intracranial blood flow caused by chronic stenosis-occlusion at the end of the internal carotid artery (ICA) (1, 2), such as neovascularization (known as “moyamoya vessels”) at the base of the brain, transdural and extracranial-intracranial collateral formation, dilation of peripheral cerebral arteries, and the development of leptomeningeal collaterals (3, 4). Besides, the conversion of the hemodynamic sources (namely, “the source of blood flow”) in the parasylvian cortical arteries (PSCAs) is also an important compensatory mechanism. PSCAs mean cortical arteries around the Sylvian fissure (SF), which not only represented the cortical vessels of anterior circulation but also were commonly selected as recipient arteries in anterior revascularization. With the development of MMD, the blood flow in PSCAs from the severe stenotic middle cerebral artery (MCA) becomes less and less. Instead, the blood flow from non-MCAs [posterior cerebral artery (PCA) or anterior cerebral artery (ACA)] starts to supply PSCAs through collateral vessels to compensate for the insufficient intracranial perfusion. Indeed, in our previous study (5), we found that only 60% (45/75) of the selected PSCAs came from MCA, rather than 100% according to the current concept. Furthermore, direct anastomoses of PSCAs with anterograde hemodynamic sources from the MCA had a high risk of postoperative CHP during STA-MCA bypass in adult patients with MMD. Thus, an accurate evaluation of the collateral flow to PSCAs will help us to better understand the spatiotemporal transformation of intracranial compensation networks in MMD as well as the selection of recipient vessels in bypass surgery.

Direct evaluation of collaterals in MMD can be archived through limited angiographic methods, including computed tomographic angiography (CTA), magnetic resonance angiography (MRA), and digital subtraction angiography (DSA). However, each of these diagnostic modalities provides specific strengths as well as limitations. DSA is considered the gold standard, but objective evaluation of collaterals is rarely performed. Collateral assessment with MRA is generally limited to proximal arterial segments at the circle of Willis. MRA velocity encoding during acquisition allows for flow-sensitive images but is constrained by anatomic resolution and is therefore only useful in proximal segments as well (6). Consequently, although cortical collaterals are essential in restoring the decreased cerebral blood flow of patients with MMD, the exact associations, connections, and territory of the arterial branches that participate in the cortical vascular network have not been objectively analyzed.

In this study, we developed a new way to analyze hemodynamic sources of PSCAs by using three-dimensional (3D) DSA and MRA fusion imaging. Through this method, we further elucidated the characteristics of blood flow sources of PSCAs in adult symptomatic MMD hemispheres in relation to the patients' demographics, Suzuki stage, and initial onset type of the hemisphere.

## Methods

### Patients

We retrospectively collected and analyzed the data of a series of adult MMD patients admitted to the hospital between March 2019 and December 2020. Regardless of whether the diagnosis was unilateral or bilateral MMD, only the symptomatic hemispheres were included in this study. A symptomatic MMD hemisphere was defined as (1) a hemisphere with a positive history of recurrent transient ischemic attack, infarction, or hemorrhage or (2) a patient with persisting neurological symptoms, such as hemiparesis, sensory deficits, or aphasia (7, 8). Clinical characteristics of the patients were recorded, including age, sex, Suzuki stage, and initial onset type (ischemic or hemorrhagic). Each patient underwent a classical DSA with 3D videos and at least a one-time MRI scan. All patients satisfied the diagnostic criteria of spontaneous occlusion of the circle of Willis as identified by the Research Committee of the Ministry of Health, Labor, and Welfare, Japan (1, 2). This study protocol was approved by the Institutional Review Board at our hospital (Kelun-2017005) and was in accordance with the Declaration of Helsinki (revised in 1983). Written informed consent was obtained from all participants.

### 3D DSA-MR fusion imaging

#### DSA

3D DSA and 2D DSA imaging were conducted on all patients using a biplane digital angiography device (Allura Xper FD20, Philips Medical Systems, Best, The Netherlands) and a trained neuroradiologist and neurosurgeon. The 2D images of bilateral ICA, external carotid artery (ECA), common carotid artery (CCA), and vertebral artery (VA) injections were routinely obtained. 3D rotational angiography was only performed after bilateral ICA injections and one VA injection. To reconstruct the 3D DSA images, mask images were also obtained. The filling run volume was reconstructed and analyzed by using a dedicated commercially available workstation (Philips Medical Systems, Best, The Netherlands).

#### MRI and MRA

Each subject underwent a high-resolution time-of-flight (TOF) MRA scan using an uMR 3.0T MRI scanner (United Imaging, Shanghai, China). The TOF-MRA scanning parameters used in this study were as follows: five slabs with 20% overlap, 30 slices per slab, repetition time = 18 ms, echo time = 3.3 ms, field of view = 22 × 22 cm^2^, flip angle = 16°, slice thickness = 0.5 mm, matrix = 512 × 512, and effective voxel = 0.39 × 0.39 × 0.5 mm^3^. The acquisition time was about 3 min and 58 s. The other imaging sequences included three-plane scout localizers, axial 2D T2-weighted fast spin echo sequence (TR/TE = 5,385/95 ms; slice thickness = 5 mm), axial 2D T2-weighted fast spin echo sequence with fluid-attenuated inversion recovery (TR/TE = 8,000/105 ms and slice thickness = 5 mm), and 3D T1 weighted fast gradient echo sequence (TR/TE = 7.2/3.1 ms, FA = 10°, resolution = 1 mm isotropic, and acquisition matrix = 240 × 256 × 256). The FOV was 22 × 20 cm^2^ on all other imaging sequences. All information regarding the images was sent to the Picture Archiving and Communication Systems (PACS, Carestream Healthcare, Rochester, NY, United States) system in the hospital.

#### 3D DSA-MRA fusion

**Figure 1:**
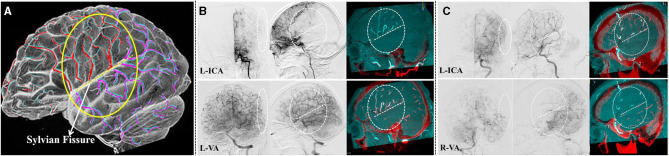
Analyzing the hemodynamic sources of the PSCAs by 3D DSA-MR fusion imaging. **(A)** The diagram of 3D DSA-MR fusion imaging used for analyzing the characteristics of collateral flow to the PSCAs. Red and purple indicate the blood on the 3D DSA images, and cyan represents the blood on the MRA images. **(B)** In this symptomatic hemisphere, all the parasylvian cortical arteries (PSCAs, in the white dashed circles and ovals) had their blood flow completely from the left posterior cerebral artery (PCA). No blood flow from the internal carotid arteries (ICAs) to the PSCAs could be found. **(C)** In this symptomatic hemisphere, the blood flow of the PSCAs below the SF clearly came from the left PCA, while that above the SF originated from the left middle cerebral artery (MCA) because the anterior cerebral artery (ACA) was occluded. From the lateral 3D DSA-MR fusion images, the collateral flow sources of the PSCAs were obviously differentiated by the Sylvian fissure (SF, white dashed line).

The obtained MRA images were imported into the DSA workstation from the PACS system. The DSA images were co-registered with the MRA images using commercially available image fusion software on the interventional tool post-processing workstation of the Philips DSA machine (Allura Xper FD20, Philips Medical Systems, Best, Netherlands). Both the MRA and DSA images were presented in different colors and displayed using volume rendering techniques (VRTs). The two 3D image volumes were overlapped to achieve fusion precision (Figure 1). The 3D DSA-MRA fusion images (axial, coronal, and sagittal views) were prepared for subsequent hemodynamic source analysis.

### Procedures for analyzing the hemodynamic sources of the PSCAs

Since PSCAs above or below SF could have different hemodynamic sources, for each patient, we analyzed the hemodynamic sources of PSCAs above/below SF separately.

By using 3D DSA-MRA fusion imaging, “the hemodynamic sources of the PSCAs” (which means the source of blood flow in PSCAs) were first identified as the ICAs or non-ICAs by two senior neurosurgeons. In the present study, ICAs were defined as a combination of MCA and anterior cerebral artery (ACA), and non-ICAs included contralateral ACA (CLA), posterior cerebral artery (PCA), and external carotid artery (ECA).

**Figure 2:**
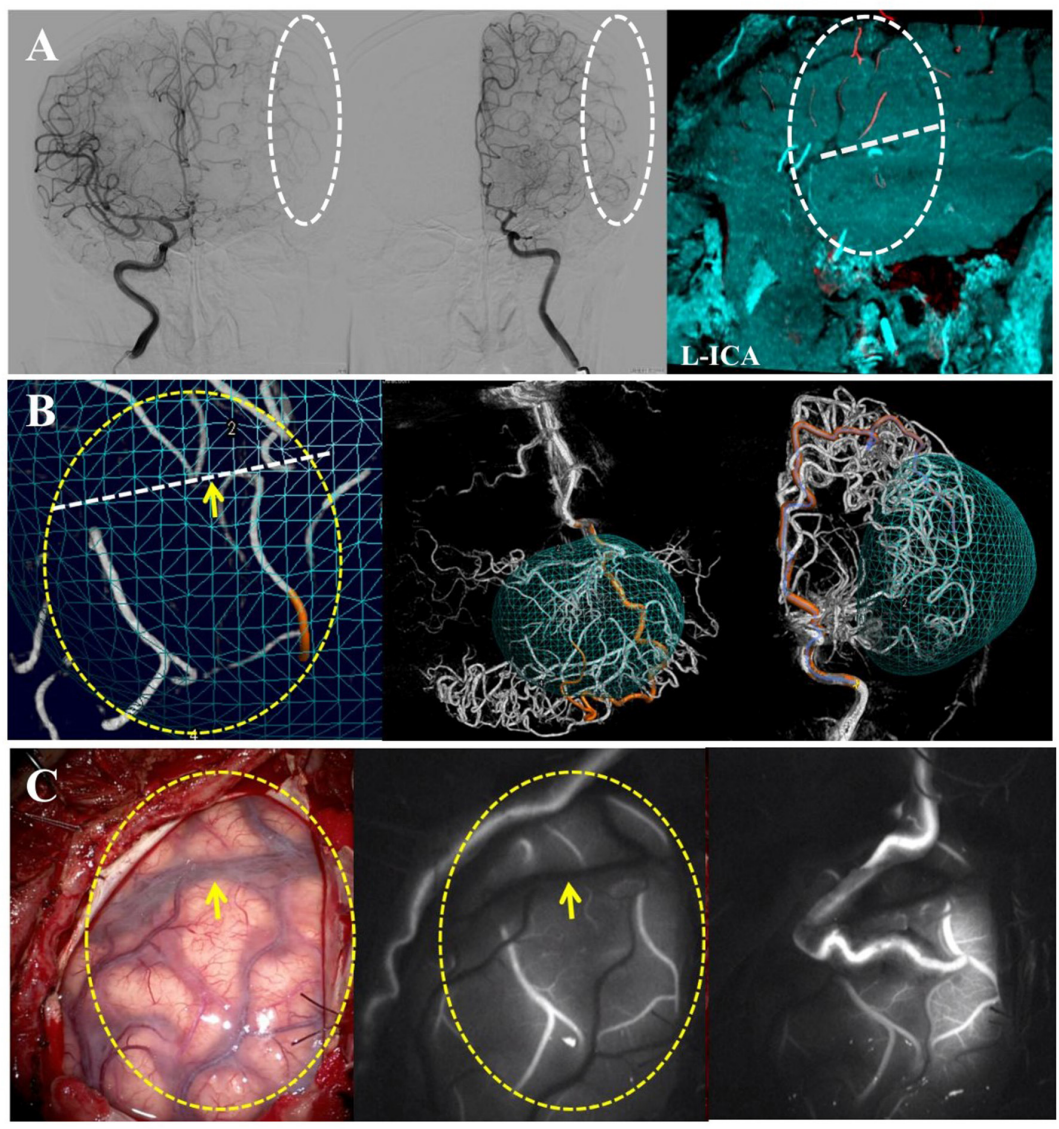
Distinguishing the hemodynamic sources of the PSCAs by an AFD analysis while difficulties encountered in the analysis procedure. **(A)** This is a unilateral MMD case with the left hemisphere involved. The 3D DSA-MR fusion images showed that the blood flow of the PSCAs above the SF might come from the ACA, CLA, or MCA. **(B)** Using automatic feeding artery detection (AFD) software, the hemodynamic source of the recipient PSCA was identified as the left ACA. If both CLA and ACA simultaneously were the flow contributions of the PSCAs, the ACA was recorded as the hemodynamic source in this study. **(C)** The intraoperative images validated our procedure for hemodynamic source analysis. In this case, the blood flow provided by the direct bypass would be widely spread into the ACA territory because of the good recipient vascular network.

When dealing with PSCAs with ICAs hemodynamic sources (whether from MCA or ACA), a built-in automatic feeding artery detection (AFD) software (Emboguide, Philips Healthcare, Best, The Netherlands) within the digital angiography unit was used (Figure 2). The AFD software procedure involved the following two simple steps: (1) manual selection of a PSCA and (2) AFD analysis, which was automatically conducted after the start position of the vessel tracking was placed on the ICA. Then, the PSCAs with ICA hemodynamic sources can be further divided into “MCA hemodynamic source” and “ACA hemodynamic source”.

The PSCAs with MCA hemodynamic source can be further divided into (1) type I: anterograde blood flow that came from the ICA via the stenotic MCA or (2) type II: anterograde blood flow that came from the ICA via “moyamoya vessels” with a lack of successful compensatory collateralization.

**Figure 3:**
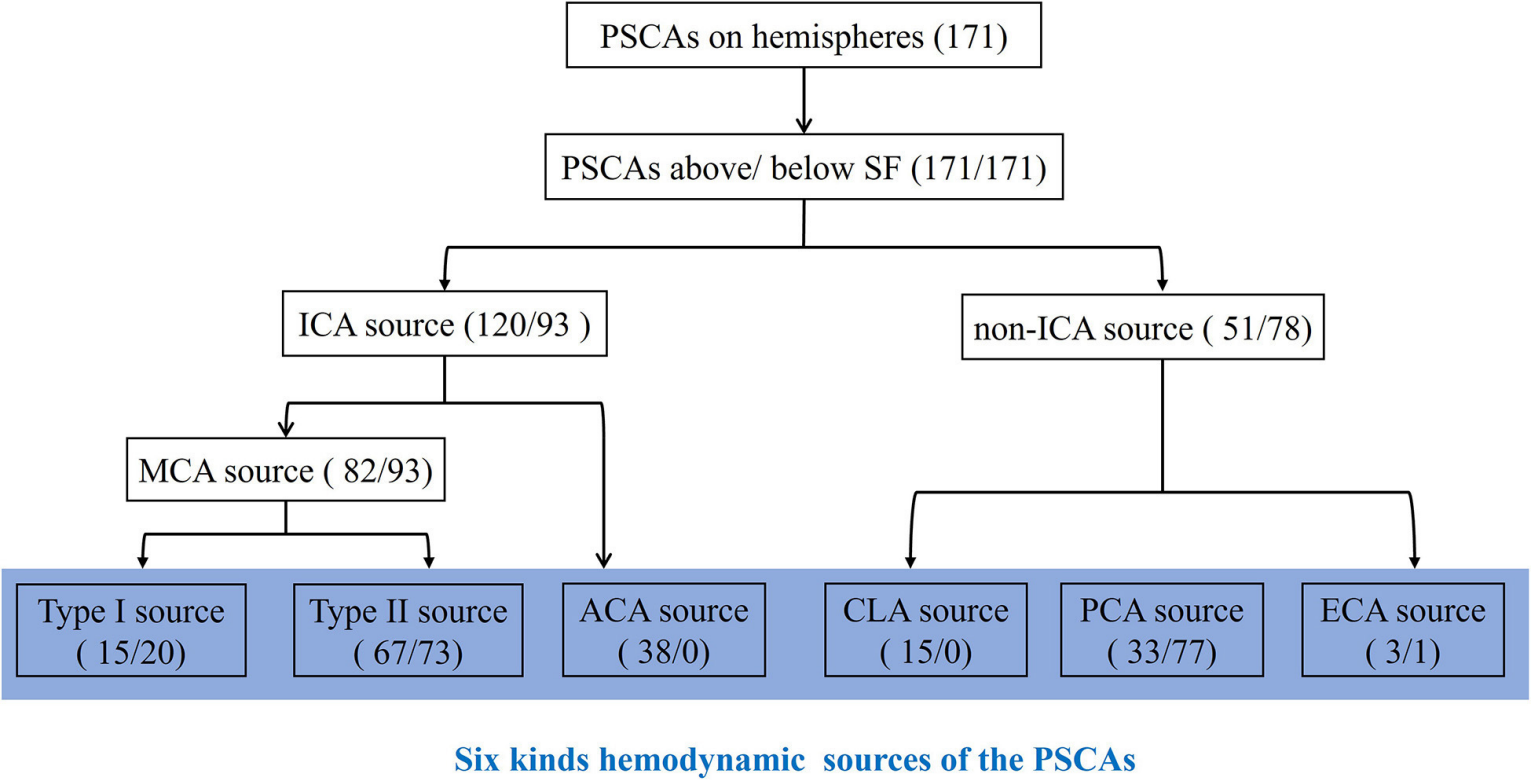
Classification and counting for hemodynamic sources of the PSCAs. For each hemisphere, the hemodynamic sources of PSCAs can be divided into two parts according to the location above or below the Sylvian fissure (SF). By using 3D DSA-MRA fusion imaging, the hemodynamic sources of the PSCAs were first identified as the ICAs or non-ICAs. ICAs were defined as a combination of the middle cerebral artery (MCA) and the anterior cerebral artery (ACA). The PSCAs with MCA hemodynamic source can be further divided into (1) type I: anterograde blood flow that came from the ICA via the stenotic MCA or (2) type II: anterograde blood flow that came from the ICA via “moyamoya vessels” with a lack of successful compensatory collateralization; non-ICAs included contralateral ACA (CLA), posterior cerebral artery (PCA), and external carotid artery (ECA). Thus, the hemodynamic sources of the PSCAs could be divided into six kinds: type I MCA, type II MCA, ACA, CLA, PCA, and ECA. The numbers in parentheses represent the number of PSCAs with corresponding hemodynamic sources. The number before the slash represents the PSCAs above the SF, and the number after the slash represents the PSCAs below the SF.

Thus, through our procedure, the hemodynamic sources of the PSCAs could be divided into six kinds: type I MCA, type II MCA, ACA, CLA, PCA, and ECA (Figure 3).

### Statistical analysis

A one-way ANOVA test was performed to check if there were any statistical differences in age and Suzuki stage between the groups. Categorical variables, such as sex and initial onset type, were analyzed in contingency tables with a chi-squared test. A multivariate statistical analysis of the factors related to the initial onset type was performed using a logistic regression model. All analyses were performed with IBM SPSS Statistics Desktop, version 24 (IBM Corp.). The results with values of *P* of < 0.05 were considered statistically significant.

## Results

### Patient demographics and hemodynamic sources of the PSCAs

A total of 171 symptomatic hemispheres from 171 consecutive adult patients (patient age range: 18–77 years; mean: 47.51 years) with MMD were enrolled in this study. In total, 86 (50.3%) were female and 85 were male patients (49.7%). A total of 57 patients had hemorrhagic onset, and 114 patients exhibited ischemic symptoms (Table 1).

**Table 1:** Baseline characteristics of included cases.

	**All patients (*n* = 171)**
Age (years) [medians (IQR)]	47.51 (18–77)
**Sex [*****n*** **(%)]**	
Female	86 (50.3)
Male	85 (49.7)
**Original side [*****n*** **(%)]**	
Right	99 (58.4)
Left	72 (41.6)
**Suzuki stage on original side [*****n*** **(%)]**	
II	15 (8.7)
III	72(42.1)
IV	63 (36.8)
V	19 (11.12)
VI	2 (1.1)
**Onset on original side [*****n*** **(%)]**	
Hemorrhagic	57 (33.3)
Ischemic	114 (66.7)
**PSCAs with ICA source [*****n*** **(%)]**	213 (62.3)
Type I MCAs source	35 (10.2)
Type II MCAs source	140 (40.9)
ACA source	38 (11.1)
**PSCAs with non-ICA source [*****n*** **(%)]**	129 (37.7)
CLA source	15 (4.4)
PCA source	110 (32.2)
ECA source	4 (1.2)

PSCAs, parasylvian cortical arteries; MCA, middle cerebral artery; ACA, anterior cerebral artery; CLA, contralateral ACA; PCA, posterior cerebral artery; ECA, external carotid artery.

As shown in Table 1 and Figure 3, there were 62.3% of PSCAs with hemodynamic sources from the ICAs, including type I MCA (10.2%), type II MCA (40.9%), and ACA (11.1%). In comparison, 37.7% of PSCAs hemodynamically originated from non-ICAs, including the CLA (4.4%), PCA (32.2%), and ECA (1.2%).

### Spatial distributional characteristics of the hemodynamic sources of the PSCAs associated with Sylvian fissures

We next evaluated the spatial distributional characteristics of the hemodynamic sources of the PSCAs around the Sylvian fissure (SF). Interestingly, we found that the hemodynamic sources of the PSCAs were obviously different above and below the SF from the 3D DSA-MRA images (*P* < 0.001) (Figures 1A, C).

**Figure 4:**
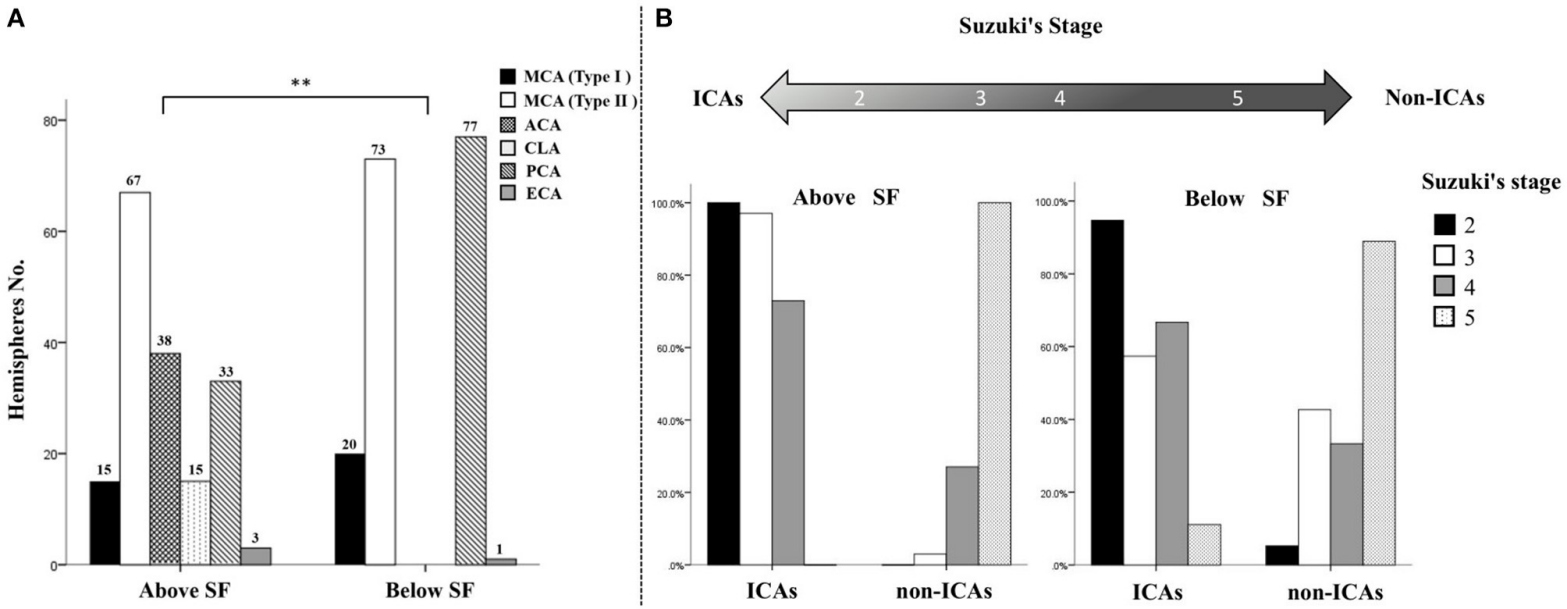
Distributional characteristics and trends of collateral flow to the PSCAs above or below the Sylvian fissure. **(A)** There was a significant difference between the hemodynamic sources of the parasylvian cortical arteries (PSCAs) above the Sylvian fissure (SF) and those of the PSCAs below the SF (**P < 0.001). All six types of hemodynamic sources in this study, including middle cerebral artery (MCA) type I (*n* = 15, 8.8%), MCA type II (*n* = 67, 39.2%), anterior cerebral artery (ACA) (*n* = 38, 22.2%), contralateral ACA (*n* = 15, 8.8%), posterior cerebral artery (PCA) (*n* = 33, 19.3%), and external cerebral artery (ECA) (*n* = 3, 1.8%) provided blood flow to the PSCAs above the SF. By contrast, the PSCAs below the SF had only four kinds of hemodynamic sources, including MCA type I (*n* = 20, 11.7%), MCA type II (*n* = 73, 42.7%), PCA (*n* = 77, 45.0%), and ECA (*n* = 1, 0.6%). **(B)** With advancing Suzuki stages, collateral flow to the PSCAs above the SF, but not below the SF, presented a significant conversion trend from internal carotid arteries (ICAs) to non-ICAs.

In PSCAs above the SF, we observed blood flow from all six kinds of hemodynamic sources, including type I MCA (15, 8.8%), type II MCA (67, 39.2%), ACA (38, 22.2%), CLA (15, 8.8%), PCA (33, 19.3%), and ECA (3, 1.8%) (Table 2; Figure 4A). However, only four kinds of hemodynamic sources were detected in PSCAs below the SF, including type I MCA (20, 11.7%), type II MCA (73, 42.7%), PCA (77, 45.0%), and ECA (1, 0.6%) (Table 3; Figure 4A).

**Table 2:** Basic characteristic analysis between the groups according to the hemodynamic sources of the PSCAs above the SF.

	**Hemodynamic sources of the PSCAs above the SF**						***P*-value**
	**MCA (type I) (*n* = 15)**	**MCA (type II) (*n* = 67)**	**ACA (*n* = 38)**	**CLA (*n* = 15)**	**PCA (*n* = 33)**	**ECA (*n* = 3)**	
Age, years	48.73 ± 9.52	46.12 ± 10.45	49.95 ± 12.11	48.93 ± 8.84	46.64 ± 11.23	44.00 ± 16.10	0.556
Male sex	7 (46.7%)	30 (44.8%)	25 (65.8%)	10 (66.7%)	12 (36.4%)	1 (33.3%)	0.108
Hemorrhagic onset	3 (20.0%)^*^	33 (49.3%)	8 (21.1%)^*^	2 (13.3%)^*^	10 (30.3%)	1 (33.3%)	0.014^*^
Suzuki's stage	2.00 ± 0.00	3.46 ± 0.51	3.00 ± 0.47	4.40 ± 0.74	4.79 ± 0.42	4.67 ± 0.58	< 0.001^*^

PSCAs, parasylvian cortical arteries; SF, Sylvian fissure; MCA, middle cerebral artery; ACA, anterior cerebral artery; CLA, contralateral ACA; PCA, posterior cerebral artery; ECA, external carotid artery.

^*^Indicates proportion of hemorrhagic onset is significantly different from that in the MCA (type II) group.

**Table 3:** Basic characteristic analysis between the groups according to the hemodynamic sources of the PSCAs below the SF.

	**Hemodynamic sources of the PSCAs below the SF**				***P*-value**
	**MCA (type I) (*n* = 20)**	**MCA (type II) (*n* = 73)**	**PCA (*n* = 77)**	**ECA (*n* = 1)**	
Age, years	47.95 ± 9.78	46.59 ± 11.45	48.29 ± 10.67	46.00 ± 0.00	0.811
Male sex	8 (40.0%)	40 (54.8%)	37 (48.1%)	0	0.461
Hemorrhagic onset	5 (25.0%)	26 (35.6%)	26 (33.8%)	0	0.728
Suzuki's stage	2.20 ± 0.62	3.52 ± 0.60	4.00 ± 0.92	5.00 ± 0.00	< 0.001^*^

PSCAs, parasylvian cortical arteries; SF, Sylvian fissure; MCA, middle cerebral artery; PCA, posterior cerebral artery; ECA, external carotid artery. ^*^Significant difference.

This phenomenon suggests that the cerebrovascular system can establish several patterns of variant collateral flows according to the special anatomical structures/microvascular networks above and below the SF to compensate for intracranial ischemia caused by ICA stenosis in MMD.

### Temporal distributional characteristics of the hemodynamic sources of the PSCAs associated with Suzuki stage

Since the patterns of variant collateral flows in PSCAs represent some kind of cerebral blood flow compensation mode in MMD, and the Suzuki stage indicates an intrinsic compensatory reorganization process, we anticipated if there was an association between the pattern of variation of collateral flow in the PSCAs and the Suzuki stage. Interestingly, we found there were significant differences between the hemodynamic sources of the PSCAs (above and below the SF) with different Suzuki stages in the ipsilateral hemisphere (*P* both < 0.001).

As shown in Figure 4B, no matter whether the PSCAs were located above or below the SF, the ICAs (MCA and ACA) were the major hemodynamic sources of the PSCAs (70.2% above and 54.4% below the SF).

In terms of PSCAs above the SF, in the hemispheres with Suzuki stage 2, the type I MCA was the primary hemodynamic source (15, 78.9%), and another source was the ACA (4, 21.1%). In the hemispheres with Suzuki stage 3, the primary blood flow sources of the PSCAs were changed to the type II MCA (36, 53.2%). Moreover, increasing blood flow from the ACA (30, 44.1%) was observed in these hemispheres. Similar to hemispheres with Suzuki stage 3, the type II MCA was still the primary hemodynamic source of the PSCAs (31, 64.6%) in the hemispheres with Suzuki stage 4. However, collateral flow from the ACA (4, 8.3%) significantly decreased, and the non-ICAs, including the CLA (5, 10.4%) and PCA (7, 14.6%), started to provide blood flow to these PSCAs. Surprisingly, in the hemispheres with Suzuki stage 5, the hemodynamic sources of the PSCAs above the SF were totally changed to the non-ICAs, including the CLA (8, 22.2%), PCA (26, 72.2%), and ECA (2, 5.6%) (Table 2).

In terms of PSCAs below the SF, the type I MCA was the primary hemodynamic source providing blood flow to the PSCAs (18, 94.7%) in Suzuki stage 2 hemispheres, and another source was PCA (1, 5.3%). In Suzuki stage 3 hemispheres, the primary blood flow sources of the PSCAs were changed to the type II MCA (39, 57.4%). Meanwhile, increasing blood flow from the PCA (29, 42.6%) was observed. In Suzuki stage 4 hemispheres, the type II MCA was still the primary hemodynamic source (30, 62.5%), and the PCA (16, 33.3%) flow was still the second collateral flow to the PSCAs below the SF but slightly decreased. In the hemispheres with Suzuki stage 5, similarly, most hemodynamic sources of the PSCAs below the SF also came from the non-ICAs, including the PCA (31, 86.1%) and ECA (1, 2.8%) (Table 3).

Together, with the advancing Suzuki stages, collateral flow to the PSCAs above the SF from the ICAs gradually decreased, while the non-ICAs significantly increased. However, the variation pattern of collateral flow to the PSCAs below the SF was not correlated to the Suzuki stage (Figure 4B).

### Association between the hemodynamic sources of the PSCAs and the initial onset type of the hemispheres

We further investigated the relationship between the hemodynamic sources of the PSCAs and the patients' basic characteristics. We found there was a significant difference in Suzuki stages between the hemodynamic sources of the PSCAs (no matter above or below SF) (both *P* < 0.001, Tables 2, 3). In various hemodynamic sources of the PSCAs above the SF, no significant difference was observed in terms of age and sex of the patients (both *P* > 0.05). However, the groups had a significant discrepancy in initial onset type (*P* = 0.014, Table 2). The result of the *Z*-test showed the proportion of hemorrhagic onset in the type II MCA group (49.3%) was significantly higher than that in the type I MCA group (20%), ACA group (21.1%), and CLA group (13.3%), respectively (Table 2). However, in the hemodynamic sources of the PSCAs below the SF group, no significant difference was observed in terms of age, sex, and initial onset type (all *P*-values > 0.05, Table 3).

The results of the multivariate analysis revealed that the initial onset type was only significantly associated with hemodynamic sources of the PSCAs above the SF [*P* = 0.026, OR, 1.541 (1.054–2.252)], rather than other factors such as age, sex, Suzuki stage, or hemodynamic sources of the PSCAs below the SF (Table 4).

**Table 4:** Multivariate analysis of factors associated with the onset type in adult symptomatic MMD hemispheres.

**Factors**	**Hemorrhagic onset (*n* = 57)**	**Ischemic onset (*n* = 114)**	**OR (95% CI)**	***P*-value**
Age, years	45.70 ± 10.52	48.41 ± 10.95	1.026 (0.995–1.057)	0.105
Male sex	27 (47.4%)	58 (50.9%)	0.883(0.459–1.698)	0.708
Suzuki stage	3.60 ± 0.84	3.59 ± 0.99	0.675 (0.397–1.149)	0.148
Hemodynamic sources of the PSCAs above the SF (type I MCA/type II MCA/ACA/CLA/PCA/ECA)	3/33/8/2/10/1	12/34/30/13/23/2	1.541 (1.054–2.252)	0.026^*^
Hemodynamic sources of the PSCAs below the SF (type I MCA/type II MCA/PCA/ECA)	5/26/26/0	15/47/51/1	0.926 (0.737–1.163)	0.509

MMD, moyamoya disease; CI, confidence interval; OR, odds ratio; PSCAs, parasylvian cortical arteries; SF, Sylvian fissure; MCA, middle cerebral artery; ACA, anterior cerebral artery; CLA, contralateral ACA; PCA, posterior cerebral artery; ECA, external carotid artery. ^*^Significant difference.

## Discussion

Cortical collateral flow is critical in MMD and has been studied by radiological angiography investigations for the past decades, including DSA, CTA, and MRA alone (6, 8–10). In our previous study, we have proven that PSCAs with different hemodynamic sources from the MCA and non-MCA had a different relationship with postoperative CHP during STA-MCA bypass in MMD (5). In this study, by using the 3D DSA-MRA fusion imaging method, we further investigated the characteristics of collateral flow to cortical vessels in MMD and their relationship with patient clinical presentations. Moreover, the present study also attempts to provide useful information in the preoperative evaluation of the recipient vascular network during STA-MCA bypass procedures for adult MMD.

As a typical chronic occlusive cerebrovascular disease, MMD represents the ultimate example of excessive collateralization, recruiting a wide range of leptomeningeal and deep parenchymal vessels (6, 11). Both collaterals may provide blood flow to the PSCAs, which are representative of the anterior cortical vessels and commonly selected as the recipient arteries in STA-MCA bypass surgeries for MMD. In the present study, we demonstrated the possibility to distinguish the blood flow sources of PSCAs by using 3D DSA-MRA fusion imaging. Previous studies have proven that the 3D DSA-MRA fusion images can provide significantly more information on the vasculature and adjacent brain tissues than the MRA/MRI or 3D DSA images alone (12–14). Consequently, by using 3D DSA-MRA fusion imaging and the AFD analysis, collateral flow to all the PSCAs in this study was identified (Figures 1, 2). Interestingly, the distributional analysis demonstrated that there was a significant difference in the hemodynamic sources of the PSCAs above and below the SF (*P* < 0.001). Furthermore, the primary collateral source that compensated for decreased MCA blood flow was the ACA above the SF and the PCA below the SF (Figure 4A). These observations are in conformity with the process of intracranial collateral recruitment, which depends on the caliber and patency of primary pathways that may rapidly compensate for decreased blood flow and the adequacy of secondary collateral routes (6).

Suzuki's angiographic staging does not represent the severity of MMD but indicates an intrinsic compensatory reorganization process and represents the physiological profile of “internal carotid (IC)-external carotid (EC)” conversion in MMD (15). This point of view is strongly supported by this current study from the perspective of trends in collateral flow to the PSCAs. With the increase in Suzuki stages, the hemodynamic sources of the PSCAs above the SF had a significant trend of conversion from ICAs to non-ICAs. However, this trend could not be observed in the hemodynamic sources of the PSCAs below the SF (Figure 4B). Different configurations may exist between collateral flow to the PSCAs above and below the SF. As demonstrated by previous studies, although the specific pathophysiological factors leading to the development of collaterals were uncertain, the arterial anatomy (anatomical factor) and diminished blood pressure in downstream vessels (demand factor) were considered critical variables (6, 16). For PSCAs above the SF, the number and size of the leptomeningeal anastomoses are greatest between ACAs and MCAs, with smaller and fewer connections between MCAs and PCAs and even less prominent terminal anastomoses between PCAs and ACAs (6). In the present study, the MCA was normally the major hemodynamic source of the PSCAs above the SF in the hemispheres with Suzuki stages 2–3 but might be subsequently changed to a relative normal ACA due to progressive stenosis of the MCA and even possibly transformed to a PCA if both MCA and ACA were occluded in the hemispheres with Suzuki stages IV–V. However, for PSCAs below the SF, the leptomeningeal collaterals are less varied and mostly limited to the connections between MCAs and PCAs. The anatomical factor was antecedent to the demand factor in the development of collateral flow to the PSCAs above the SF and just opposite in the formation of collateral flow to the PSCAs below the SF. This discrepancy might be the reason why the trend of conversion from ICAs to non-ICAs was hard to observe in the hemodynamic sources of the PSCAs below the SF.

In adult MMD patients, the development of thalamic and/or choroidal collaterals is a predictor of hemorrhagic events (17–21). This finding is supported by our discovery that the hemispheres with hemodynamic sources of the PSCAs above the SF from the type II MCA had a relatively high risk of hemorrhagic stroke when compared to those from the type I MCA, ACA, and CLA, which more frequently suffered cerebral ischemia (Table 2). The multivariate analysis further revealed that only the hemodynamic sources of the PSCAs above the SF were significantly associated with the onset type (*P* = 0.026, Table 4). In this study, the type II MCA source represented anterograde blood flow that came from the ICA via the moyamoya vessels, including thalamic and/or choroidal collaterals, to the cortical arteries and a lack of successful compensatory collateralization. In such a situation, the abundant abnormal vascular anastomoses at the base of the brain may experience heavy blood flow, thus resulting in vessel dilatation, formation of microaneurysms, and vessel rupture (5). However, we observed that collateral flow to the PSCAs above the SF from the ACA or CLA was significantly correlated to the ischemic symptoms, which may be due to the blood flow pressure of the abnormal vascular anastomosis, which is significantly released by blood flowing into the relatively normal and unobstructed ACA or CLA. These findings may contribute in predicting the types of episodes in an asymptomatic MMD hemisphere.

### The potential significance of the findings in STA-MCA bypass surgeries for MMD

As it has been shown in previous studies, the craniotomy planned for most STA-MCA bypass cases is most frequently centered around the SF (22, 23), and the PSCAs are usually selected as the recipient arteries for direct anastomoses. The results of this study demonstrated that, although located in an operating field, the PSCAs above and below the SF might have different hemodynamic sources. If we performed direct anastomosis on a recipient PSCA with anterograde blood flow from the type II MCA, the poor cortical vasculature of such PSCA could result in limited flow distribution after direct revascularization and a high risk of postoperative hyperperfusion (5, 24–26). Consequently, avoiding the selection of a recipient PSCA with its blood flow from the type II MCA is suggested when performing direct bypass surgeries for patients with MMD.

### Limitations

This study has several limitations. First, it is a single-center study, which creates a regional bias in the sample, which could be an issue. Second, as only the symptomatic hemispheres that underwent bypass surgeries were included, data on hemispheres with Suzuki stages 1 and 6 were absent in this study. Third, visualization of cortical arteries was not better than visualization of stenosis of proximal arteries from the TOF MR angiography. Fortunately, this limitation was compromised by the use of 3D DSA imaging. Both methods worked in a mutually beneficial manner in the analysis of the hemodynamic sources of the PSCAs.

## Conclusion

The hemodynamic sources of PSCAs can be accurately identified by 3D DSA-MRA fusion imaging and then help to understand the quality of the recipient vascular network before STA-MCA bypasses MMD. We demonstrated that the collateral flow sources of the PSCAs above the SF were more varied when compared to those below the SF and revealed the ICAs to non-ICAs conversion with advancing Suzuki stages. We also demonstrated that the hemispheres with hemodynamic sources of the PSCAs above the SF from the type II MCA had a relatively high risk of hemorrhagic stroke, and those from the type I MCA, ACA, and CLA more frequently presented with ischemic symptoms. This classification of the hemodynamic sources may assist with figuring out the complex parasylvian cortical collateral flow and its relationship with clinical presentations and postoperative CHP in MMD.

## Data availability statement

The raw data supporting the conclusions of this article will be made available by the authors, without undue reservation.

## Ethics statement

The studies involving human participants were reviewed and approved by the Institutional Review Board (IRB) at Zhongnan Hospital of Wuhan University (approval number: Kelun-2017005). Written informed consents were waived since all identifiable personal details has been hidden.

## Author contributions

MH: methodology, validation, investigation, and writing—original draft preparation. JY: methodology, validation, formal analysis, investigation, writing—original draft preparation, and visualization. JZ: conceptualization, formal analysis, investigation, resources, supervision, writing—review and editing, and funding acquisition. JC: conceptualization, resources, supervision, writing—review and editing, and funding acquisition. All authors contributed to the article and approved the submitted version.
